# Identification of heterozygous mutations of ABCC8 gene responsible for maturity-onset diabetes of the young with exome sequencing

**DOI:** 10.1007/s00592-024-02410-1

**Published:** 2024-11-18

**Authors:** Yanxia Liu, Shuxin Ren, Chaofeng Zhu, Sufang Chen, Huijuan Zhang, Juan Zhang, Jianhua Li, Yanyan Jiang

**Affiliations:** 1 Department of Endocrinology and Metabolism, The First Affiliated Hospital of Zhengzhou University, Zhengzhou, China; 2 Genetic and Prenatal Diagnosis Center, the First Affiliated Hospital of Zhengzhou University, Zhengzhou, China; 3 Department of Geriatric Endocrinology, The First Affiliated Hospital of Zhengzhou University, Zhengzhou, China; 4 Institute of Monogenic Disease, School of Medicine, Huanghuai University, Zhumadian, China; 5 Department of Emergency Medicine, The First Affiliated Hospital of Zhengzhou University, Zhengzhou, China

**Keywords:** ABCC8 gene, Mutation, Maturity-onset diabetes of the young, Exome sequencing, Precision medicine

## Abstract

**Background:**

Although the MODY12 subtype, caused by *ABCC8* mutations, is rare, it is highly sensitive to sulfonylureas. The identification of *ABCC8* mutations in patients clinically diagnosed with MODY has the ability to contribute to the precise management of diabetes.

**Methods:**

Genetic analysis of two families with MODY were conducted using whole-exome sequencing (WES) and Sanger sequencing. The spatial structures of the mutant proteins were constructed using MODELLER and PyMOL software to provide further evidence of pathogenicity.

**Results:**

The heterozygous missense mutations V357I and R1393H in *ABCC8* were found in probands of two unrelated MODY pedigrees, which co-segregated with the hyperglycemic phenotypes in these two pedigrees. Detection of the V357I mutation enabled the proband of family A to successfully transfer from insulin to sulfonylurea (SU). After 3 months of follow-up for the SU trial, the HbA1c level of proband A improved from 12.4% at the initial diagnosis to 7.20%. Proband B was treated with insulin because of pregnancy and poor islet function. In silico analysis indicated that the R1393H mutation resulted in a longer hydrogen bond distance to L1389 and cleavage of carbon-hydrogen bonds to V1395, A1390, and L1389.

**Conclusions:**

We have described two pathogenic missense mutations in *ABCC8* in Chinese families with MODY. Our findings support the heterogeneity in the clinical features of MODY12 caused by *ABCC8* mutations.

**Supplementary Information:**

The online version contains supplementary material available at 10.1007/s00592-024-02410-1.

## Introduction

The ATP-sensitive potassium (K_ATP_) channel, constituted by the pancreatic β-cell sulfonylurea receptor (SUR1) and inward-rectifying potassium channel subunit Kir6.2, is a pivotal modulator of insulin release [1]. Activating mutations in the ABCC8 gene, which encodes the SUR1 protein, have previously been described as a cause of neonatal diabetes or maturity-onset diabetes of the young (MODY) [2, 3], whereas inactivating mutations in this gene usually lead to hyperinsulinemia hypoglycemia (HI) in infancy [4, 5]. Furthermore, patients with HI who carry dominantly inherited *ABCC8* mutations occasionally develop diabetes later in life [6, 7]. Although more than 1000 mutations scattered throughout *ABCC8* have been discovered to trigger hypo-or hyperglycemia, ABCC-related MODY is relatively rare (http://www.hgmd.cf.ac.uk/ac/index.php).

MODY, an autosomal dominant inherited diabetes, is characterized by the onset of hyperglycemia at an early age (classically before the age of 25 years) and impaired insulin secretion with minimal or no defects in insulin action [8]. More than 80% of patients with MODY have been previously misdiagnosed with type 1 or type 2 diabetes because of the atypical clinical characteristics and the low availability of genetic testing, which hinders early preventative management and precision treatment based on genetic etiology [9, 10]. Variants in at least 13 genes have been identified as being responsible for MODY, of which heterozygous ABCC8 mutations are a cause of MODY12 [3, 8]. MODY12 patients exhibit variable clinical features with impaired glucose tolerance (IGT), overt diabetes, or insulin-dependent diabetes from a young age to adulthood. Most patients can be successfully transferred to low-dose sulfonylurea (SU), and even the defective capacity of insulin secretion is recovered after SU treatment in some individuals [3, 11]. Therefore, despite its rare occurrence, the detection of ABCC8-realted MODY by genetic testing may help target patients for SU therapy.

In the present study, we have described two heterozygous missense mutations in *ABCC8* in clinically defined MODY probands using exome sequencing and conducted a pedigree co-segregation study. Additionally, the tertiary structure of the mutant protein was constructed using the MODELLER and PyMOL software to provide further evidence of pathogenicity.

## Materials and methods

### Subjects

We have studied two unrelated probands from Chinese families with clinically defined MODY based on the following criteria: age of onset of diabetes < 25 years, first-degree relative with diabetes, and negative for glutamic acid decarboxylase (GAD) and insulinoma-associated antigen-2 (IA-2) antibodies. First-degree relatives of the probands and the affected family members were incorporated in the study for mutation detection. Written informed consent was obtained from all the participants. This study was approved by the Ethics Review Committee of First Affiliated Hospital of Zhengzhou University. American Diabetes Association (ADA) criteria (2023) were used to diagnose diabetes, impaired fasting glucose (IFG), and IGT [8].

### Genetic analysis

Genomic DNA was extracted from peripheral blood leukocytes or saliva using standard procedures. WES was performed on clinically defined MODY probands using the SureSelect Human All Exon V5 Enrichment kit (Agilent, USA) on an Illumina HiSeq 4000 platform (Illumina, USA). Burrows-Wheeler Aligner (BWA) was used to map the sequencing reads to the human reference genome (hg19/GRCh37), and the GATK v3.3.0 best practice was applied to detect single nucleotide polymorphisms and small insertions or deletions. Candidate variants were screened according to the results of harmfulness prediction and conservative analysis, and the inheritance pattern of the pedigree and gene function were used for further analysis.

Sanger sequencing was conducted on the two probands in order to verify the results of exome sequencing and their affected relatives for co-segregation studies. Fragments containing the identified *ABCC8* mutations were amplified. The purified polymerase chain reaction products were sequenced using an ABI3730 capillary sequencer (Applied Biosystems, Foster City, CA, USA).

### Assessment of clinical data

The clinical features of the affected individuals carrying *ABCC8* mutations, including age at onset, presentation of diabetes, and treatments for diabetes, were evaluated. The parameters for diabetes in the probands with *ABCC8* mutations were obtained from a thorough clinical examination of their medical records. Standard oral glucose tolerance testing (OGTT) was conducted in two probands to measure the insulin secretory response when referred and after a 3-month follow-up of hypoglycemic therapy. As previously described, HOMA-IR and HOMA-B were calculated in order to assess insulin resistance and pancreatic β-cell function in the probands [12]. Diabetic peripheral neuropathy was assessed by the SUDOSCAN device by measuring electrochemical skin conductance, and sudomotor dysfunction was defined as hands and feet electrochemical skin conductance (FESC) < 60 µs.

### In silico analysis of identified variants

The possible functional significance of the identified missense variants was evaluated in silico using prediction software, including SIFT, PolyPhen-2, LRT, MutationTaster, FATHMM, and CADD. Specific regions of the SUR1 protein encoded by *ABCC8* from different species were aligned using MutationTaster (https://www.mutationtaster.org/) to evaluate the conservation across species. MODELLER10.4, the most frequently employed tool for 3D model building in homology modeling, was used to construct the protein structures of wild-type and mutated SUR1, and the protein model was further optimized and also evaluated [13]. PyMOL software (https://sourceforge.net/projects/pymol/) and Discovery Studio2019 were used to analyze the changes in three-dimensional and two-dimensional structures caused by mutations, respectively.

## Results

### Exome sequencing identified the V357I and R1393H mutations in *ABCC8*

**Fig. 1:**
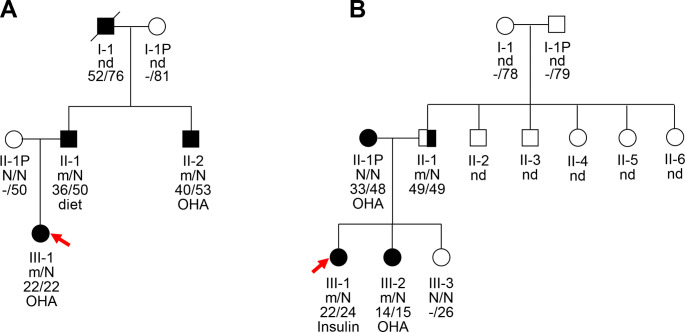
Pedigrees, genotypes, and clinical characteristics of the two MODY12 families. Black circles and squares, participants diagnosed with MODY12; white circles and squares, normal glucose tolerance (NGT); red arrows, the probands for the two families. The numbers under the symbols are the family members’ identification numbers, followed by the genotype of mutation, then age at diagnosis of diabetes and age at examination, followed by treatment for diabetes. nd = not detected; OHA = oral hypoglycaemic agents; N = normal allele; m = mutant allele

**Fig. 2:**
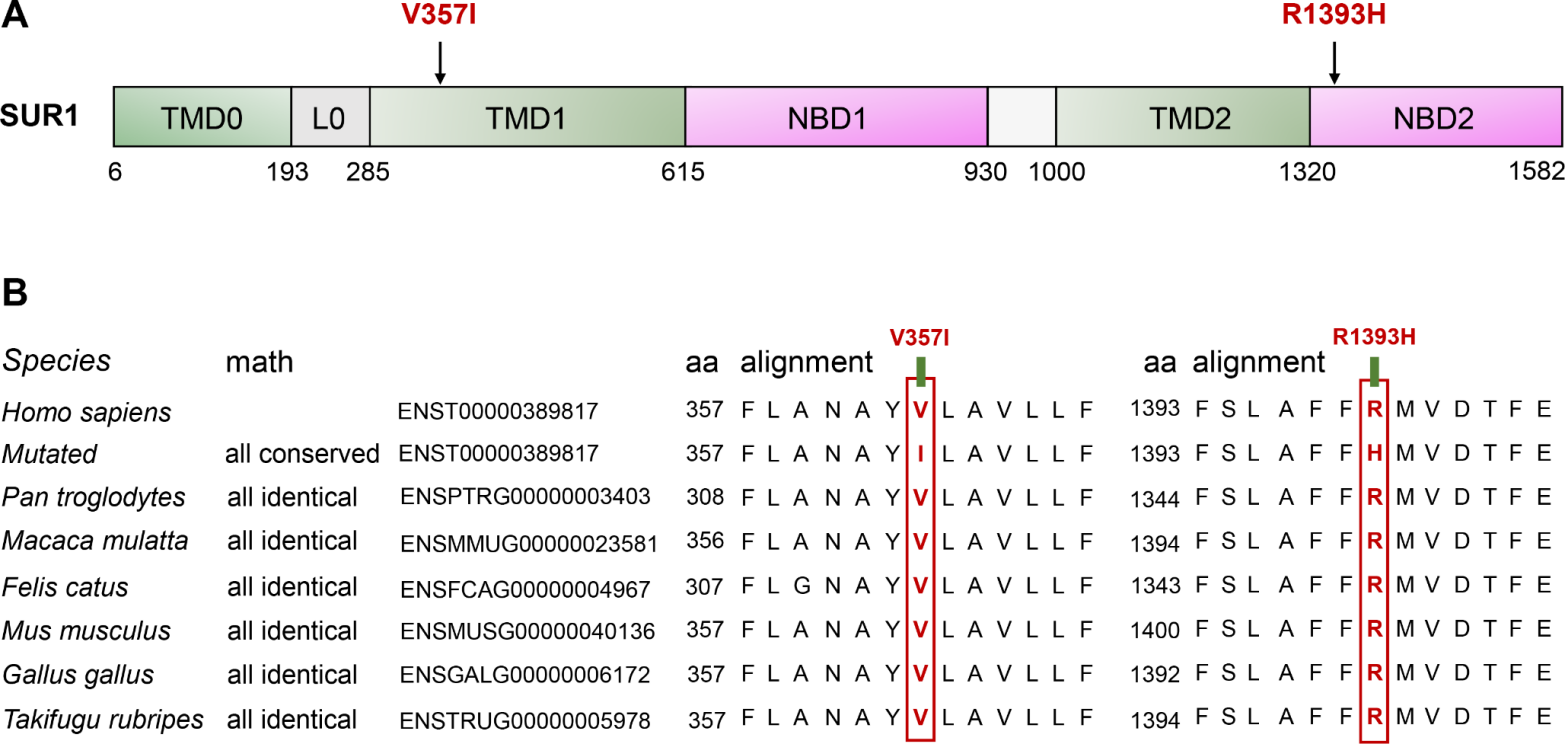
Schematic illustration of the SUR1 protein encoded by ABCC8 gene and the conservation analysis of the mutation sites. **A**. The distribution of identified mutations among the linear SUR1 protein. The functional domains of the SUR1 protein were shown; Filled arrows indicated the mutations identified in *ABCC8.* TMD, transmembrane domain; NBD, nucleotide-binding domain. **B.** Alignment of specific regions of SUR1 protein from different species

Heterozygous missense mutations in the ABCC8 gene (NM_000352.6), V357I (c.1069G > A, p.Val357Ile), and R1393H (c.4178G > A, p.Arg1393His), were identified in the probands of two unrelated MODY pedigrees, which co-segregated with the hyperglycemic phenotypes in these two pedigree cases (Fig. 1). Both mutations affected highly conserved residues within the functional domain of *ABCC8*, and the amino acid substitutions within these sites may be pathogenic (Fig. 2). Additionally, the results from multiple software packages predicted the probable damaging effects of the V357I and R1393H mutations in *ABCC8*, further supporting their pathogenicity.

### Clinical features of MODY families with *ABCC8* mutations

The clinical phenotypes and biochemical parameters of the probands from the two identified MODY12 families at referral are shown in Table 1 (Table 1). In family A, four individuals were diagnosed with overt diabetes, and the earliest diagnosed age was 22 years (Fig. 1). The proband, aged 22 years, was diagnosed with diabetes due to hyperglycemia, with a fasting plasma glucose (FPG) level of 6.1 mmol/L and glycosylated hemoglobin (HbA1c) level of 12.4% on physical examination. Due to poor pancreatic β-cell function (fasting C-peptide 0.63 ng/mL), she was prescribed insulin pump therapy. In addition, the patient also had fatty liver, and diabetic peripheral neuropathy. Owing to the young age at onset of the proband and her father and uncle, WES was conducted on the proband, resulting in the identification of the V357I mutation in *ABCC8*. Furthermore, this mutation was also found in her affected father and uncle, but not in her euglycemic mother, indicating that this mutation co-segregated with the hyperglycemia phenotype in this family. By considering the effective response to sulfonylurea in patients with ABCC8-related diabetes, the proband was tentatively switched to glimepiride (2 mg/day). HbA1c levels improved to 7.20% and *the fasting C peptide was 0.71 ng/ml (elevated 18%) after a 3-month glimepiride trial*. The father of proband A had been on a diet due to mild hyperglycemia since his diagnosis of diabetes. The proband’s uncle was well controlled with metformin in combination with dietary therapy. The paternal grandfather of proband A was diagnosed with obvious diabetes at age 52 years and died of esophageal cancer at age 76 years, and we were unable to obtain his DNA sample.

**Table 1:** Clinical features and biochemical parameters of the probands in the two families

	Proband 1	Proband 2
Age (year)	22	24
Onset age (year)	22	22
BMI (kg/m^2^)	23.62	24.03
Birth weight (kg)	3.2	3.8
Gestational age at birth (weeks)	39	40
History of neonatal hypoglycemia	no	no
FPG (mmol/l)	6.1	7.92
2 h-PG (mmol/l)	14.8	12.21
FINS (uU/mL)	2.3	2.43
2 h-INS (uU/mL)	7.5	1.90
Fasting C-peptide (ng/ml)	0.63	1.35
2 h C-peptide (ng/ml)	1.98	1.11
HbA1c (%)	12.4	7.40
HOMA-IR	0.62	0.86
HOMA-B (%)	17.69	11.00
TC (mmol/L)	4.75	4.91
TG (mmol/L)	1.45	1.34
HDL-C (mmol/L)	0.86	1.05
LDL-C (mmol/L)	3.52	3.43
Antidiabetic therapy	Sulfonylureas	Insulin

BMI, Body mass index; FPG, fasting plasma glucose; 2 h-PG, 2 h plasma glucose; FINS, fasting insulin; 2 h-INS, 2 h insulin; HbA1c, glycated hemoglobin; HOMA-IR, homeostasis model assessment of insulin resistance; HOMA-B, homeostasis model assessment of β-cell function. TC, total cholesterol; TG, triglyceride; HDL-C, high-density lipoprotein cholesterol; LDL-C, low-density lipoprotein cholesterol

The R1393H mutation in proband B occurred within the nucleotide-binding domain 2 of SUR1, a previously reported hotspot domain for dominantly acting mutations. Proband B was the first child of a 25-year-old woman with a full-term natural delivery at week 40 of an uneventful pregnancy and a birth weight of 3.8Kg, in the absence of neonatal hypoglycemia. This proband had a normal BMI, was diagnosed with IGT at the age of 22 years, and developed overt diabetes one year later. The proband currently had multiple diabetic complications, including retinopathy, nephropathy, neuropathy, and cardiac insufficiency. *Due to the presence of multiple complications*,* insulin therapy was prescribed. Upon re-evaluation of pancreatic islet function after a three-month follow-up*,* no significant changes were observed.* The L992Sfs5* mutation in *PKD1* (NM_001009944.3; c.2764del; p. L992Sfs*5) of the proband screened by WES, inherited from her mother, may contribute to the abnormal renal function, and both the proband and her mother had radiographically confirmed polycystic kidney (Supplementary Fig. 1). The mutation was also found in her sister aged 15 who was diagnosed with diabetes at the age of 14 years. Nevertheless, the R1393H mutation of these two sisters was inherited from their father, presenting with glucose intolerance rather than the diabetic mother who developed diabetes at age 33 years. The maternal grandmother of proband B, who suffered from diabetes, died at the age of 50 years, and the parents of the proband’s father had normal glucose tolerance; however, their DNA was not available to check for co-segregation in this family.

### Construction of protein models for the wild type and mutant SUR1 proteins

**Fig. 3:**
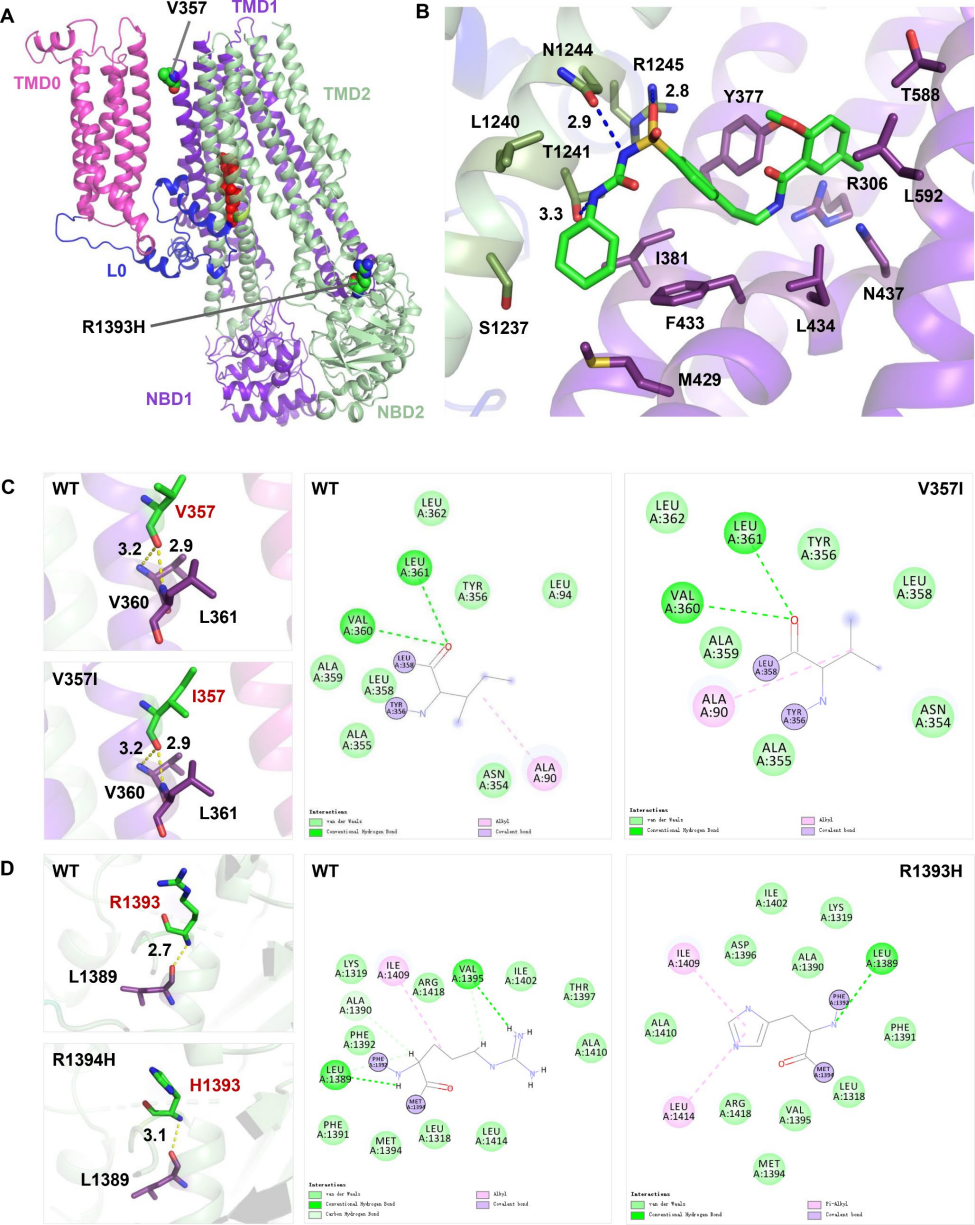
Molecular modelling of V357I and R1393H mutation of SUR1. **(A)** Location of the two mutations and the glibenclamide binding site in the three-dimensional model of human SUR1 protein. **(B)** A close-up of the glibenclamide binding pocket in SUR1 protein and the residues that immediately interact with glibenclamide. **(C)** Three-dimensional and two-dimensional structures of wild type and mutant V357I. **(D)** Three-dimensional and two-dimensional structures of wild type and mutant R1393H

In order to further analyze the structural abnormalities of proteins caused by the mutations, we constructed protein models of wild-type SUR1 and MODY12-associated mutants (V357I and R1393H). A Ramachandran plot constructed using MODELLER indicated that 99.8% of the amino acid residues in the SUR1 protein had reasonable dihedral angles (Supplementary Fig. 2). The locations of the two *ABCC8* mutations and the glibenclamide-binding sites were mapped to the global landscape of SUR1 (Fig. 3A). A close-up of the glibenclamide-binding pocket in the SUR1 protein and the residues that immediately interact with glibenclamide are shown in Fig. 3B. The two mutations identified in this study were not included in the binding pocket (Fig. 3B). The V357I mutation is located in the TMD1 domain, and introduces a methyl group without altering the polarity and charge of the amino acid. Further spatial structure analysis revealed that the V357I mutation did not alter interactions with the surrounding amino acids (Fig. 3C). Although the R1393H mutation located in the NBD2 domain did not change the polarity and charge of the amino acid, it introduced an imidazolyl moiety and had a smaller molecular weight. Analysis on the three-dimensional structure found that the hydrogen bond distance between wild-type R1393 and L1389 was 2.7 Å, which was extended to 3.1 Å after mutation (Fig. 3D). Further analysis of the two-dimensional interactions around the amino acids using Discovery Studio2019 found that wild-type R1393 formed carbon-hydrogen bonds with the surrounding amino acids V1395, A1390, and L1389, and these interactions disappeared when mutated (Fig. 3D).

## Discussion

MODY12 is a rare subtype of MODY. In this study, we have detected two previously reported mutations, V357I and R1393H, in *ABCC8* from two families with clinically suspected MODY [1, 14]. V357 mutation has been described separately in patients with HI and late-onset diabetes, but genetic evidence from both parents is lacking [1]. The V357I mutation of proband A was derived from his affected father, and although we were unable to obtain DNA from his deceased diabetic grandfather for co-segregation verification, we also detected this mutation in the uncle of the proband. These results supported the diabetogenic effects of the V357I mutation (Fig. 1A). Notably, the R1393H mutation has been found in patients with PHHI, and functional analysis has supported the inactivation property of R1393H because this mutation produces no functional K_ATP_ channels [5, 15]. While *ABCC8* inactivation mutations typically cause a hypoglycemic phenotype, some dominant inherited mutations may lead to the development of diabetes in adulthood, supporting the hyperglycemic phenotype observed in proband B [16, 17]. However, proband B and her sister did not report a history of neonatal hypoglycemia, and it is possible that they had undetected CHI. Several factors may explain the variability in the clinical phenotypes. The ABCC8-R1393H mutation may not be the only cause of diabetes, since the father of the proband carrying this mutation only showed glucose intolerance, whereas the proband’s mother without the mutation was diagnosed with diabetes at 33 years of age. In addition, other modifiers, such as diabetes susceptibility genes inherited from his mother and superimposed environmental factors, may have promoted early onset diabetes in proband B [11]. A recent study showed that the same ABCC8 genotype can present a biphasic phenotype in the same patient or a reverse phenotype in the same pedigree, further supporting the phenotypic variability caused by the same ABCC8 mutation [18, 19]. We have also identified the PKD1-L992Sfs5* mutation in proband B and her mother. PKD1 is the most common pathogenic gene in autosomal dominant polycystic kidney disease (ADPKD) and PKD1 mutations have recently been recognized as biomarkers of ADPKD that can predict renal prognosis [20]. The shorter duration of diabetes and earlier renal dysfunction in proband B may be partly attributed to the PKD1 mutations in her mother, who also had diabetic nephropathy. Additionally, patients with PKD1 mutations show a more significant decrease in renal function and worse prognosis than those without non-PKD1 mutations [20].

The SUR1 protein, encoded by the gene ABCC8, contains three transmembrane domains (TMD0, TMD1, and TMD2), two cytoplasmic nucleotide-binding domains (NBD1 and NBD2), and a long cytoplasmic loop (L0) that connects to the ATP-binding cassette (ABC) core (Fig. 3A) [21, 22]. V357 is located at the extracellular end of the second transmembrane helix in TMD1, with the side chain pointing toward the outside of the helix (Fig. 3A). In silico analysis showed that V357 was not involved in binding to sulfonylureas, and that the V357I mutation did not change the spatial conformation of the mutated protein (Fig. 3B and C). The previously reported Y356C mutation, occurring at adjacent sites in V357, also did not change the charge and polarity of amino acids; however, in vitro experiments have confirmed that the mutation can reduce the ATP sensitivity of K_ATP_ channels and glucose-stimulated Ca2^+^ influx, thus leading to the development of adult diabetes [23]. It is possible that the V357I mutation causes the hyperglycemic phenotype of the mutation carriers in family A through a similar mechanism, which requires further functional studies for confirmation. R1393H occurs in NBD2, which binds to MgADP and is critical for KATP channel activation; in the presence of Mg-nucleotides, sulfonylureas can inhibit channel activity by preventing dimerization of NBD2 [21, 24]. The alteration of spatial conformation may lead to dysfunction of the mutant, as previous studies have confirmed that the co-expression of the SUR1-R1394H mutant with Kir6.2 produced no functional channels, which is related to a trafficking disorder influencing the retention of mutant proteins in the trans-Golgi network [5, 15].

The high sensitivity of ABCC8-related MODY12 and neonatal diabetes to sulfonylurea has enabled some patients to switch from insulin to sulfonylurea [2, 3]. Moreover, follow-up studies have also shown that even after a diabetes course of up to 10 years, some patients with MODY12 and NDM maintain good metabolic control with sulfonylurea [25, 26]. This is because sulfonylureas can bypass genetic defects caused by *ABCC8* mutations by binding to SUR1 and closing K_ATP_ channels in the pancreas, thereby promoting endogenous insulin secretion [1]. The excellent glycemic control achieved by sulfonylurea therapy and better quality of life due to the withdrawal of insulin injections highlight the need for precise treatment of patients with ABCC8 mutations [27]. Proband A in our study also successfully switched from insulin to glimepiride after identification of ABCC8-V357I mutation. Her carrier relatives can achieve acceptable glycemic control with only strict lifestyle interventions owing to mild hyperglycemia, whereas sulfonylureas can be used as first-line oral hypoglycemic agents as the disease progresses. However, proband B received insulin because of pregnancy, poor islet function and severe complications.

In summary, we have identified two ABCC8 mutations in patients with clinically suspected MODY. Family co-segregation is prone to support the diabetes-causing effect of V357I, whereas the loss-of-function mutation R1393H results in adult diabetes without a history of hypoglycemia in infancy, suggesting heterogeneity in the clinical features of patients with MODY12. However, whether MODY12 or adult diabetes developed from CHI are both monogenic diabetes. Identifying monogenic diabetes is clinically important, as it can guide precise treatment and help detect relatives with MODY.

## Supplementary Information

Below is the link to the electronic supplementary material.

Supplementary Material 1

Supplementary Material 2

Supplementary Material 3

## Data Availability

The original contributions of this study are available publicly. These data can be found in the ClinVar database (https://www.ncbi.nlm.nih.gov/clinvar/).
