# Supplementary material for: Identification of heterozygous mutations of ABCC8 gene responsible for maturity-onset diabetes of the young with exome sequencing
Source: Acta Diabetol. 2024 Nov 18;62(6):935–42. doi: 10.1007/s00592-024-02410-1 (PMC12141373; doi:10.1007/s00592-024-02410-1)
Supplement: Supplementary file 1 — Supplementary Material 1 [file 592_2024_2410_MOESM1_ESM.docx]

Supplementary Material

Identification of heterozygous mutations of ABCC8 gene responsible for maturity-onset diabetes of the young with exome sequencing

**Yanxia Liu^1^†, Shuxin Ren^1^†, Chaofeng Zhu^2^, Sufang Chen^3^, Huijuan Zhang^1^, Juan Zhang^4^, Jianhua Li ^5^#, Yanyan Jiang^3^#**

*** Correspondence:** Jianhua Li 823096796@qq.com, Yanyan Jiang jylijh@163.com

# Supplementary Figures


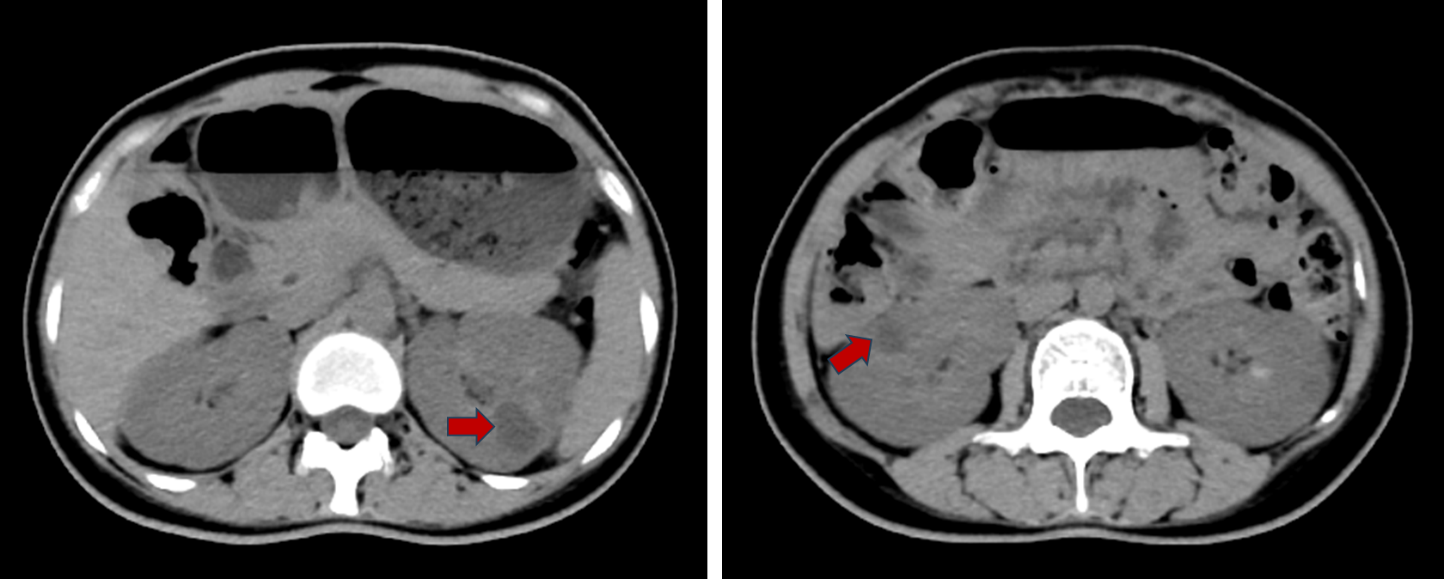


**Supplementary Figure 1.** MRI imaging of kidney cyst in proband B.


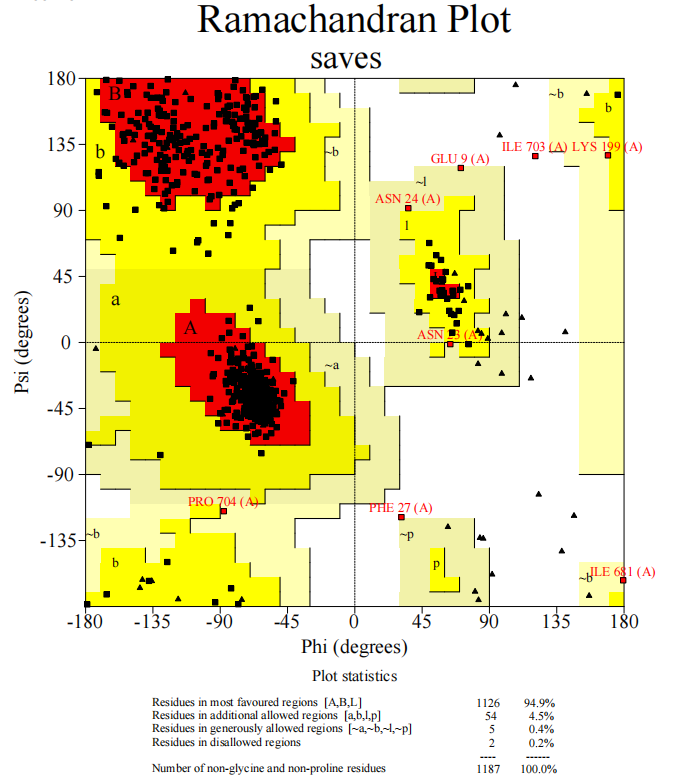


**Supplementary Figure 2.** The calculated Raman map of SUR1 protein model evaluation obtained by MODELLER. 94.9% of residues in most favored regions [A, B, L]. 4.5% of residues in additional allowed regions [a, b, l, p]. 0.4% of residues in generously allowed regions [~a, ~b, ~l, ~p]. 0.2% of residues in disallowed regions. Number of non-glycine and non-proline residues is 100%.
