# Supplementary material for: Identification of heterozygous mutations of ABCC8 gene responsible for maturity-onset diabetes of the young with exome sequencing
Source: Acta Diabetol. 2024 Nov 18;62(6):935–42. doi: 10.1007/s00592-024-02410-1 (PMC12141373; doi:10.1007/s00592-024-02410-1)
Supplement: Supplementary file 2 — Supplementary Material 2 [file 592_2024_2410_MOESM2_ESM.pdf]

郑州大学第一附属医院
血糖观察表

科别： 内分泌科二　　姓名： 易金萍　　性别：女　　年龄：22岁　　床号： +47　　住院号： ZY010004607867

诊断： 糖尿病伴有神经的并发症；　　单位：mmol/L

日期	早餐		中餐		晚餐		睡前	凌晨	备注
	餐前	餐后2小时	餐前	餐后2小时	餐前	餐后2小时			
	签名	签名	签名	签名	签名	签名	签名	签名	
2022-06-27					15.1	23.6	16.8		
					刘静娟	石婉君	石婉君		
2022-06-28	OGTT	检查	15.1	14.2		7.5	5.8	8.5	0'7.6吴红婧　120'16.4 张瑜
	贾萧燕	张瑜	石婉君	许亚平		王妮娅	王妮娅	贾萧燕	
2022-06-29	8.2	14.7	12.4	未进食	7.9	11.0	9.0	6.7	
	崔圳	贾萧燕	贾萧燕	吴红婧	张瑜	许亚平	许亚平	崔圳	
2022-06-30	7.7	14.4	6.4	12.0	9.4	7.8	6.0	5.9	
	王妮娅	张瑜	张瑜	贾萧燕	刘静娟	孙文雅	孙文雅	王妮娅	
2022-07-01	6.2	6.7	6.2	7.7	7.7	6.6	6.3	6.0	
	石婉君	贾萧燕	张瑜	贾萧燕	许亚平	王妮娅	许亚平	石婉君	
2022-07-02	OGTT	检查	11.7	5.4	5.9	检查	6.7	6.2	0'6.1 张瑜　30' 6.5 张瑜 60' 9.8 张瑜<br>120'14.8 张瑜　180'14.9 张瑜
	吴红婧	张瑜	张瑜	石婉君	崔圳	王妮娅	孙文雅	吴红婧	
2022-07-03	6.1	6.4	9.0	13.1	6.1	5.3	6.4	7.2	
	许亚平	石婉君	崔圳	石婉君	吴红婧	王婉君	贾萧燕	许亚平	
2022-07-04	5.8	7.3	5.4	4.7	6.3	5.3	5.1	5.3	
	孙文雅	张瑜	吴红婧	石婉君	贾萧燕	贾萧燕	许亚平	孙文雅	
2022-07-05	6.5	6.4	9.4	7.1				5.0	
	崔圳	贾萧燕	张瑜	贾萧燕				崔圳	
