# Supplementary material for: Identification of heterozygous mutations of ABCC8 gene responsible for maturity-onset diabetes of the young with exome sequencing
Source: Acta Diabetol. 2024 Nov 18;62(6):935–42. doi: 10.1007/s00592-024-02410-1 (PMC12141373; doi:10.1007/s00592-024-02410-1)
Supplement: Supplementary file 3 — Supplementary Material 3 [file 592_2024_2410_MOESM3_ESM.docx]

Conflict of Interest Form

The authors have no relevant financial or non-financial interests to disclose.
